# The relationship between game genre, monetization strategy and symptoms of gaming disorder in a clinical sample of adolescents

**DOI:** 10.48101/ujms.v129.10386

**Published:** 2024-03-06

**Authors:** Frida André, Per Bore, Theo Toresson, Mitchell Andersson, Emma Claesdotter-Knutsson

**Affiliations:** a Lund University, Faculty of Medicine, Department of Clinical Sciences Lund, Section for Psychiatry, Lund, Sweden; b Region Skåne, Malmö Addiction Center, Clinical Addiction Research Unit, Malmö, Sweden; c Save the Children, Sweden, Gothenborg; d Region Skåne, Child and Adolescent Psychiatry, Regional Outpatient Care, Lund University Hospital, Lund, Sweden

**Keywords:** Gaming disorder, game genre, microtransactions, loot boxes, monetization

## Abstract

**Background:**

Gaming disorder (GD) has been introduced as a new diagnosis in the International Classification of Disease 11 (ICD-11). Currently, there’s limited understanding of how various video games may differentially contribute to the risk of developing GD. The main aim of this study was to examine the relationship between individuals’ game genre preferences, their preferred games’ monetization strategies, and GD Symptoms.

**Methods:**

A total of 85 patients undergoing treatment for GD at a child and youth psychiatric clinic were included in the study. Their preferred games were classified into five novel genres based on gameplay similarities and objectives, and further categorized based on their monetization strategy.

**Results:**

Symptom burden of GD, measured with Game Addiction Scale for Adolescents (GASA), was highest for those playing Free-to-Play (F2P) games and lowest for Pay-to-Play (P2P) players. Players of Competitive Games endorsed higher GD symptom burden, whereas players of Story-driven games reported lower GD symptom burden. Symptoms of GD were associated with attention-deficit hyperactivity disorder (ADHD) diagnosis in males.

**Conclusions:**

This study reveals that game genre preference is influenced by sex, age, and certain psychiatric diagnoses. The categorizing of games into genres is increasingly complex and our research introduces a novel categorization in a developing research field. The result of this study suggests that the monetization model is important to consider while trying to understand the relationship between game characteristics and GD symptoms.

## Introduction

Video gaming is a common leisure activity, with 68% of 13–16 year olds and 55% of 17–18 year olds in Sweden reporting that they play daily (1). For most people, it is a positive activity, which can enhance mental health and overall well-being (2, 3). However, for some individuals, gaming can become problematic. As of 2019, the International Classification of Diseases and Related Health Problems (ICD-11) recognizes gaming disorder (GD) as an official diagnosis (4). GD is defined by a gaming behavior characterized by impaired control, increased priority given to gaming over other activities, continuation, or escalation of gaming despite negative consequences, as well as gaming resulting in distress or impaired functioning in daily life (4). The field of GD research is still in its infancy, yet there is an increasing demand for more studies seeking to improve our understanding of its etiology and manifestation. One research gap that has been highlighted is how GD, game types, and structural game characteristics are related (5).

The prevalence rate of GD varies across studies, likely due to the use of different measures and cross-cultural disparities, with notably higher prevalence in Asian countries (6). A recent meta-analysis showed a global prevalence rate of 1.96% (7). Much is still unknown regarding why some people develop GD and some people do not. The two most prominent risk-factors in research are younger age and male sex (7). Although extant evidence suggests that gaming experiences and motivation may differ by sex, few investigations have specifically sought to determine how gender affects gaming behavior and experiences. (8, 9). Studies have shown high comorbidity with other psychiatric disorders such as depression, anxiety, and obsessive-compulsive disorder (10–12), as well as neuropsychiatric conditions such as attention-deficit hyperactivity disorder (ADHD) and autism spectrum disorder (ASD) (13, 14). Specifically, ADHD seem to be highly linked to GD (10). ADHD is identified by persistent and disruptive manifestations of inattention, hyperactivity, and impulsivity (15) Inattentive symptoms have been showed to be reciprocally associated with GD (16) indicating that the condition both predisposes to and is aggravated by GD. ASD is marked by enduring deficits in social communication and a consistent, repetitive display of behaviors, interests, or activities (15). It has been hypothesized that individuals with ASD, who often exhibit heightened attention to detail, weak central coherence, and sometimes specific visual skills, might possess a certain adaptability for video game tasks that demand swift visual scanning in intricate environments (17). Also, the difficulties inherent to these neuropsychiatric conditions can conceivably contribute to an appearance of the challenges presented in video games as more enjoyable, manageable, or rewarding than other tasks (17).

People with GD have a higher risk of suicidal ideation, sleep disturbance, emotional deregulation, poorer executive functioning, higher impulsivity, and poorer academic performance (16, 18–21). The motives for gaming have been identified as important predictors of GD. Studies have shown that the use of gaming as an escape from normal lives and gaming as a way of coping in particular are related to more problematic gaming behavior (22).

The video game industry has evolved immensely since its inception, and the games it produces have also undergone substantial transformation. The relationship between GD and specific games or game genres is sparsely researched (23, 24) and the research that has been performed is partly dated as the games that have been studied in some cases have changed their genre belonging (25). As the field of video games has developed, games have borrowed characteristics and functions from other games and genres, making it increasingly difficult to categorize games into existing genres (23, 26). Today, a multitude of game genres coexist, with many overlapping with one another, making it increasingly common that new games are being classified under multiple genres. To investigate whether specific game types are more prone to trigger GD, attempts have been made to examine the potential correlation between game genres and GD symptom burden. However, this is a challenging endeavor as categorizing a game into a specific genre may not be a valid measure for games that incorporate elements from various genres, making direct comparison with other games, whether they are within the same genre or in a different one, complex. The most common video game genres are: role-playing games (RPG), action and adventure games, strategy games, simulation games, first-person shooter (FPS), sport games, massively multiplayer online role-playing game (MMORPG), multiplayer online battle arena (MOBA), and sandbox and battle royale games. In Rehbein and colleagues systematic review of the association between game genre and symptoms of GD, they extracted 32 articles about game genre and problematic gaming behavior (24). They found that MMORPGs and FPS games were most strongly associated with symptoms of GD, but recent studies also highlight that there may be an association between playing MOBA games and endorsing greater GD symptom burden (24). MOBA is a relatively new and increasingly popular game genre, which would explain why this association has only recently been identified. Rehbein et al., also mentions two studies on MOBA games whom neither reported a significant association between the genre and GD symptoms (24). In summary, research suggests that playing games from certain genres may contribute to a higher burden of GD symptoms. However, as new games are released and game genres continue to evolve, ongoing research is necessary to assess the addictive potential of these emerging titles.

Monetary elements in games as well as the financial principles driving them have received immense attention and criticism (27–30). The knowledge on how different monetization strategies affect the risk of developing problematic gaming behavior is insufficient (27). Pay-to-Play (P2P) video games are a category of video games where players are required to make an upfront payment to purchase the game before they can access and play it. Free-to-Play (F2P) video games on the other hand are made available to players at no initial cost, allowing them to download and play the game for free. F2P games generate revenue through various in-game monetization strategies rather than relying on an upfront purchase fee. But many P2P games also rely on in-game monetization to increase their revenue. In-game monetization strategies take on many forms, including advertising and microtransactions. Examples of microtransactions include, loot-boxes, cosmetic customization, pay-to-win, power-ups, in-game currency, etc. (27–30). A distinction can be drawn between microtransactions, where you receive an object that you choose yourself, and loot-boxes, which give random outcomes akin to gambling (29). More and more research has been focused on investigating similarities between microtransactions and gambling behavior (28). Studies have shown that individuals with higher in-game expenditure are more likely to endorse symptoms of problematic gaming behavior, problem gambling, and psychological distress (30, 31).

Firstly, the aim of this study was to investigate the impact of video game genre combined with monetization model on GD symptoms. Recognizing the complex nature of video game genres, where many games belong to multiple categories, this study attempts to simplify the categorization by merging game genres.

Secondly, we were interested in how the game genre preference may depend on sex, age, and psychiatric diagnosis.

To our knowledge, no previous study explores both gaming genres, video game monetization, and their potential links to GD.

## Methods

### Participants

This study is an extension of a randomized control trial (RCT) with the aim to determine the effectiveness of relapse prevention as a treatment for IGD. The study was performed within three different child and adolescent psychiatric (CAP) units in Region Skåne, Sweden from the 1st of September 2021 to the 30th of December 2022 (32). In total, 113 patients participated in the RCT and each of them were asked to name the games they played most often, with a maximum of three games. The data used for this study were collected at baseline, prior to the intervention. Only those who answered that question were included in this study, which totaled 85 participants (75%) of whom 63 were male and 22 were female with a mean age of 14.28 years (SD = 1.37).

### Measures

In addition to assessment regarding gaming behavior and the mentioning of favorite games, basic demographics routinely recorded in the journal were collected, such as sex, age, and diagnosis.

### Game Addiction Scale for Adolescents

The Game Addiction Scale for Adolescents (GASA) was used to screen for GD (33). GASA applies to gaming behavior during the past 6 months and is one of the most frequently used measures for GD (34, 35). The instrument is based on the DSM-V criteria for problem gambling (salience, tolerance, mood modification, relapse, withdrawal, conflicts, and problems; (15, 33). Responses are given on a 5-point scale from 1 = never to 5 = very often. An item is considered endorsed when rated 3 or higher (33). DSM-V suggests that half of the criteria should be met to qualify for a diagnosis, which concordantly was our cut off for inclusion in the study (15).

### Video game category by genre

In this study, we have used a novel approach to categorize video games into genres. The purpose is to simplify the genre classification that also reflects the actual differences between different games and why people play them. At the core, we have started from classic genres, such as MMORPG, FPS, etc., and then simplified by combining several genres. The aim was to categorize the games based on similarities in how they are played. The categorization of the games was done by a group of six adults who regularly play video games out of which four were licensed psychologists and one was a game developer, and five genres were created.

The five genres included:

1. ‘Competitive games’, consisting of genres such as MOBA, FPS, RTS (real-time strategy) and Sports games. The games have a competitive focus and are played in shorter matches with friends or random players.
2. ‘Story-Driven games’ genre is played offline, and the games have a distinct beginning and end. These games come from the classic genres as Action-Adventure games and RPGs.
3. ‘Single Player Simulation Strategic Progression’ (SimStrat). These games are predominantly a solo experience, but they differ from the Story-driven games group in that they do not have a clear narrative and ending. The focus is for the player to create their own world or to find the best strategy to navigate through the game.
4. ‘MMO Extended’ (MMOE) consists of classic MMO games as well as Survival games, Sandbox games, and Co-op games. What these games have in common is a stronger social component where you often play with the same players over a longer period of time (in open worlds or in limited sessions).
5. ‘Casual games’. These are limited games without a narrative focus, typically consisting of one type of challenge that increases in difficulty, such as platformers and other casual games.

### Video game category by model of financing

The games were also categorized by financial model (how the developers monetize the games), as illustrated in Table 1. Three financial models were created: One time cost (P2P), One time cost with microtransactions, and F2P. An illustration of how popular games were categorized is presented in Table 1.

**Table 1:** Games by genre and financial model.

Financial model	Game genre				
	Competitive	Story-driven	Simstrat	MMOE	Casual
Free-to-Play	Apex Legends Call of Duty Clash Royale Counter Strike Fortnite League of Legends Overwatch Valorant Agario Arena of Valor Warface		Hayday Sims TocaBoca Call of War	Roblox Rival Stars Destiny Genshin Impact Star Stable	GeoGuessr Will Hero Geometry Dash
Pay-to-Play	Forza Dead by Daylight Super Smash Bros Escape from Tarkov Assetto Corsa FIFA* NBA 2K* Rainbow Six*	Pokémon Horizon Dark Souls Hitman Fallout	Civilization Hearts of Iron Stardew Valley Trainz People playground Rock of Ages Snowrunner Rouge	ARK Dungeon Defenders Generation Zero Minecraft* Garry’s Mod* Rust* Grand Theft Auto* Red Dead Redemption* Sea of Thieves* World of Warcraft*	

Popular games stratified by monetization strategy and genre. MMOE, massively multiplayer online extended, Simstrat, Single Player Simulation Strategic Progression.

* indicates one-time cost (P2P) with available microtransactions.

### Data preparation

Statistical analyses were performed in SPSS (IBM SPSS statistics version 29). Sex, age (> 15 years and ≤15 years) and GD diagnosis were transformed into binary variables. The least prevalent diagnoses were merged into a new variable labelled ‘other diagnosis’ (see Table 2). This variable included depressive disorders (mild depressive episode, unspecified depressive episode) anxiety disorders (anxiety disorder, unspecified, mixed anxiety and depressive disorder, generalized anxiety disorder), obsessive compulsive disorder, adjustment disorder, other symptoms and signs involving emotional state, pathological gambling, and diagnoses primarily used during the psychiatric evaluation phase (observation for suspected mental and behavioral disorders, general psychiatric examination, not elsewhere classified, examination and observation for unspecified reason, observation following alleged rape or seduction, examination and observation for unspecified reason). The GASA score was summed and used as a continuous value with a minimum of seven points to a maximum of 35.

**Table 2:** First column: descriptive statistics. Column 2–6: Fishers exact test for comparisons of genre representation within sex-, age-, and main psychiatric diagnosis.

	Total sample (*n* = 85)	Competitive^1^ (*n* = 59)	Story driven^2^ (*n* = 7)	SimStrat^3^ (*n* = 14)	MMOE^4^ (*n* = 49)	Casual^5^ (*n* = 3)
Sex, % (*n*)						
Male	74.1 (63)	76.2 (48)	6.3 (4)	15.9 (10)	55.6 (35)	1.6 (1)
Female	25.9 (22)	50.0 (11)	13.6 (3)	18.2 (4)	63.6 (14)	9.1 (2)
*P*		0.031	0.368	0.750	0.619	0.63
Age, % (*n*)						
≤15	80.0 (68)	63.2 (43)	8.8 (6)	19.1 (13)	60.3 (41)	4.4 (3)
>15	20.0 (17)	94.1 (16)	5.9 (1)	5.9 (1)	47.1 (8)	0.0 (0)
*P*		0.017	1.000	0.283	0.413	-
ADHD, % (*n*)						
Yes	40.0 (34)	67.6 (23)	0.0 (0)	29.4 (10)	67.6 (23)	0.0 (0)
No	60 (51)	70.6 (36)	13.7 (7)	7.4 (4)	51.0 (26)	5.9 (3)
*P*		0.813	-	0.015	0.179	-
ADD, % (*n*)						
Yes	15.3 (13)	69.2 (9)	0.0 (0)	7.7 (1)	69.2 (9)	0.0 (0)
No	84.7 (72)	69.4 (50)	9.7 (7)	18.1 (13)	55.6 (40)	4.2 (3)
*P*		1.000	-	0.685	0.543	-
ASD, % (*n*)						
Yes	10.6 (9)	55.6 (5)	33.3 (3)	11.1 (1)	33.3 (3)	22.2 (2)
No	89.4 (76)	71.1 (54)	5.3 (4)	17.1 (13)	60.5 (46)	1.3 (1)
*P*		0.446	0.024	1.000	0.159	0.029
Other Dx, % (*n*)						
Yes	34.1 (29)	75.9 (22)	13.8 (4)	6.9 (2)	48.3 (14)	3.4 (1)
No	65.9 (56)	66.1 (37)	5.4 (3)	21.4 (12)	62.5 (35)	3.6 (2)
*P*		0.459	0.223	0.125	0.250	1.000

MMOE, massively multiplayer online extended; Simstrat, Single Player Simulation Strategic Progression; Dx, Diagnosis.

The participants were sorted into binary genre categories and financing model categories based on the games they mentioned (see Table 1). Some participants mentioned games belonging to multiple genres or financing models, which meant that the variables could not be constructed mutually exclusive.

### Data analysis

The mean GASA score was estimated for each gaming category (genre and financing model).

Sample demographics (sex, age, other diagnosis) within gaming genre were calculated using crosstab and differences were tested using Fisher’s exact tests.

Results of the Fisher’s exact tests were used to select variables to be included in the linear regression with GASA scores as the dependent variable. The variables that showed a significant association to a game genre, were used in the linear regression analysis together with the most popular genres. We chose to include the P2P variable in the regression model because our hypothesis was that this category would differ distinctly from the other two. All statistical analyses were conducted in SPSS (IBM SPSS statistics version 29).

## Results

Sample characteristics are shown in the first column in Table 2. Our final sample consisted of 85 participants, of whom roughly 25% were female. A majority were aged 13–15 years old and the mean age was 14.28 years. ADHD was the most common diagnosis, followed by ADD and ASD. The most preferred genres were Competitive games and MMOE, among both male and female participants.

### Genre, GASA score and associated variables

Table 2, column 2–4 presents the representation of sex, age, and psychiatric diagnosis within the game genres. Male participants and older age were significantly overrepresented within the Competitive genre. ADHD was significantly underrepresented among Story-driven gamers and significantly overrepresented among SimStrat-gamers. ASD was significantly overrepresented among both Story driven gamers and Casual gamers.

### Financial model and GASA score

**Figure 1:**
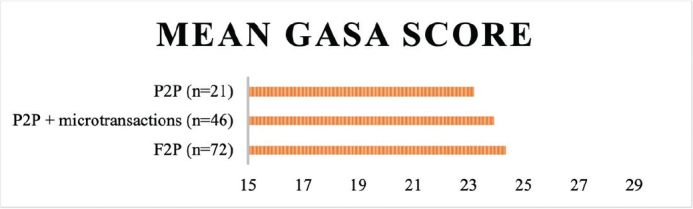
Mean GASA score by financial model. P2P, Pay to play; F2P, Free to play.

As shown in Figure 1, the mean GASA score tended to be higher among the participants who reported F2P games and increasingly low as the financial element’s decrees.

**Figure 2:**
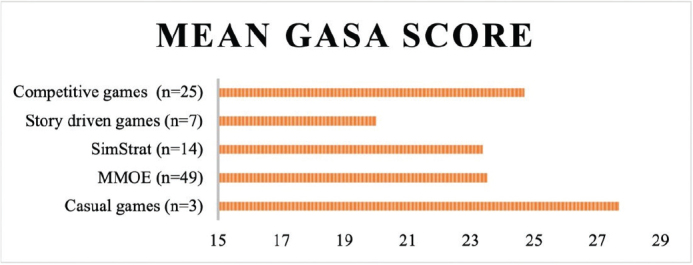
Descriptive: Mean GASA score by game genres.

Figure 2 illustrates how the mean GASA score varies between game genres. The few individuals who played casual games and the greater number individuals who played competitive games seem to display high GASA scores relative to those who preferred story driven games.

### Financial model, genre, diagnosis and mean GASA score

A multiple regression was conducted to predict GASA scores from demographics, game genre, and financial model variables. Our model significantly predicted GASA t-scores, accounting for 24.9% of the overall variance, F(7,77) = 3.655, *P* = 0.002, *R*^2^ = 0.249, adj. *R*^2^ = 0.181. Regression coefficients and standard errors are presented in Table 3. Male sex (*P* = 0.03) and ADHD (*P* = 0.03) were found to be positively associated with GASA score, whereas being 15 years or older (*P* = 0.07), MMOE gaming (*P* = 0.02) and P2P gaming (*P* = 0.03) were negatively associated with GASA score.

**Table 3:** Linear regression: Dependent variable GASA T-score.

	Unstandardized coefficients			Standardized coefficients	
	*B*	Std error	95% CI for *B*	Beta	Sig.
Male sex	5.452	2.377	0.719 – 10.185	0.240	0.025
Age >15	-4.990	2.670	-10.307 – 0.327	-0.201	0.065
ADHD	4.851	2.179	0.513 – 9.190	0.239	0.029
ASD	1.153	3.492	-5.800 – 8.106	0.036	0.742
Competitive games	1.189	2.465	-3.721 – 6.098	0.055	0.631
MMOE	-5.203	2.253	-9.690 – -0.716	-0.259	0.024
P2P	-5.317	2.386	-10.068 – -0.566	-0.231	0.025

Std, Standard; CI, Confidence interval; ADHD, attention-deficit hyperactivity disorder; ASD, autism spectrum disorder; MMOE, massively multiplayer online extended; P2P, Pay-to-play.

## Discussion

The world of online gaming is in constant transformation and the categorizing of games into genres is increasingly complex. Several of the most popular games are multi-genre, and therefore they target different groups of gamers, which play the games differently (e.g. competitive, social, exploratory, creative). Monetization strategies are unambiguous and accounting for them, we can better understand the addictive components in games. Firstly, the aim of this study was to explore whether there was an association between the preferred game genre and/or the financial model of the game and the severity of GD symptoms. Secondly, we wanted to explore whether there were differences in the characteristics of the gamers within the different genres, in terms of sex, age and/or psychiatric diagnosis.

The most preferred genres were Competitive and MMOE games, with the most common monetization strategy being F2P. We present figures showing a tendency toward highest GD symptoms among the casual and competitive gamers and among the F2P gamers. Males and those being older than 15 years were significantly overrepresented within the competitive genre. The Story driven genre showed an overrepresentation of ASD, whereas ADHD was overrepresented among the SimStrat gamers. The regression analysis showed male sex and ADHD were positively associated with GASA scores, whereas being 15 years or older, MMOE gaming and P2P gaming were negatively associated with GASA scores.

Casual gamers presented with high GASA scores, although few participants were included in this group. The games listed in this genre were exclusively F2P games and one could speculate whether the tendency could be explained by characteristics of the genre or by elements caused by the monetization strategy.

Competitive gamers showed relatively high GASA-scores. Some of the games included in this genre have previously been classified as FPS (Overwatch, Counter Strike) and RTS (League of Legends, Clash Royale), genres that have been associated with particularly high GD symptoms (35). Kim et al. (35) showed that RTS and FPS gamers showed significantly higher levels of both symptoms of tolerance and withdrawal than other genres. The Competitive genre included games that have several features in common, most notably, the competitive element. The majority have a ranking ladder, which gives the player clear feedback on their achievements on a regular basis in relation to the gaming population. Rewards of achievement have previously been described as an underlying factor of GD (36). Possibly, the ranking mechanism contributes to a potential of playing more than intended, thus increasing the risk of problematic gaming behavior. Many players describe the frustration of quitting a gaming session on a ‘losing streak’, and the wish to always end with a win (36). This, in combination with other features, such as social pressure, a ‘fear of missing out’, features like the ‘first win of the day’ or similar features intended to create a habit of logging in, as well as eliciting a sunk cost fallacy of having invested a lot of money, may contribute to the higher GASA-scores (36, 37). However, the regression analysis showed no association between Competitive games and GD symptoms.

The male gamers were overrepresented within the Competitive games genre. Sex differences in genre preferences are poorly researched but there are some results to be found, which are consistent with this specific finding. Fernandez et al., showed that males preferred competitive genres; such as action-shooters, sport, fight and strategy game whereas females preferred nonviolent and occasional genres such as social simulation, and brain and skill games (8). On the other hand, Laconi et al., showed that competition as a motive for gaming was a relevant predictor for female GD (38), and probably the reason for the female underrepresentation within this genre is multifactorial.

The Story driven games group presented the lowest GASA-scores in this sample. The fact that these games do not include social elements commonly seen in F2P games might contribute to a relatively low GASA-score. Social features in games have been mentioned as an element with pathological potential (36). However, the Story driven games genre only includes P2P games, and an alternative explanation could be that as these games provide a guaranteed income, they are not as dependent on other financing elements and therefore they might be less focused on mechanisms designed to maintain and retain the customers’ gaming behavior, to guarantee a payoff.

The regression analysis showed that ADHD was positively associated with high GASA score. ADHD has consistently been linked to GD, and the DSM-V even lists ADHD as a comorbidity of GD (10, 13). No association was seen between ASD and GD symptoms. One could speculate that the number of individuals with ASD might have been too few to detect a significant association between ASD and GD. ASD and GD have also been reported as associated but the relationship appears less consistent across studies (39).

The dividing of the sample into categories based on different monetization strategies showed an interesting pattern. The mean GASA score tended to be higher among the participants who reported F2P games and lower among P2P gamers. The in-between group of participants playing P2P games with microtransaction presented a mean GASA score just below the F2P gamers. The difference in GASA score should not be exaggerated as mean differences may not be statistically significant. However, there is an overlap between the groups, and one could argue that the differences probably would have been greater if the categories were mutually exclusive. In accordance with this speculation, the regression analysis showed that P2P gaming was negatively associated with high GASA score.

There is a lack of knowledge regarding how different monetization strategies affects the risk of developing problematic gaming behavior (27). A study by Dreier and colleagues shows that F2P games are highly associated with problematic gaming behavior, a finding consistent with our own results (40). A possible hypothesis is that since F2P games are free to download, but monetize based on player engagement duration and are structured to keep players playing as long as possible. Examples of design patterns observed in F2P games include having a daily bonus for returning to the game or for the first win of the day, daily quests, offering advantages to purchasing players (pay-to-win), utilizing the sunk cost fallacy (given that gamers may have invested significant financial and time resources) and loot-boxes or random drop rewards and similar gambling like features (41, 42). It could be argued that all these strategies aim to cultivate a habit where players spend both time and, consequently, money in the game. Björk et al. argued for the phrase ‘dark game design patterns’ defined as ‘a pattern used intentionally by a game creator to cause negative experiences for players, which are against their best interests and likely to happen without their consent’ (43). These elements aim to push the player toward spending more time, money, or social capital than they intended, often leaving the player feeling regret or that time was wasted. Some games utilize real life social obligations toward friends and family, as the players will fall behind in their progress if they abstain from playing the game for too long (43).

## Limitations

The presented findings should be considered in the light of the study’s limitations. One obvious limitation of this study is the sample size. The relatively small number of participants requires certain adaptations of selected analyzes such as a limitation of the number of variables to include in our regression analysis. We wanted to explore our potential associations among male and female gamers separately and we based our choice of variables, to include in the regression analysis, on prior testing of sample characteristics and therefore we do believe that we were able to capture the most important associations in this sample. However, it would have been interesting to dig deeper into some areas such as the association between GD symptoms and not only genres but specific titles. Some of the games within this sample were represented only by one individual and for both ethical and practical reasons we did not want to make further specifications.

The study is performed in a clinical sample of adolescents recruited to participate in an RCT aimed to evaluate a treatment for GD. One could argue that this fact could reduce the generalizability of our results. However, as GD is highly associated to child psychiatric conditions such as ADHD, and as GD has been shown to be specifically common within the CAP context, it is relevant to further explore aspects of the behavior within this specific setting.

Also, our attempt to simplify the categorization of games into genres based on similarities regarding how the games are played, could be considered a limitation as this fact complicates comparison with previous research. However, the field of research on gaming genres is evolving and previous research is far from consistent in terms of how to classify specific titles. Games of today are increasingly multifaceted and can be played in various ways, which make any classification system arbitrarily and new perspectives of both the games and the playing thereof is probably conductive for further understanding.

The participants in this study listed the games they played most often at the time the survey was taken and therefore the gaming behavior is not captured in its full complexity. Possibly, some individuals who spend a lot of their time gaming have one main game that they return to while playing other titles when they are released or return to them for a couple of months when a new expansion is added. This could hypothetically also mean that the high GASA-scores seen in the competitive games group is an illustration of how gamers with problematic gaming behavior quickly work their way through the titles they are interested in, and then congregate in these games (rather than the fact that the games in themselves are more addictive).

Also, unfortunately our categories are not mutually exclusive, neither the monetization categories nor the genre categories. The overlap between categories might cloud true associations. However, this fact captures the reality that most gamers engage in games from separate genres and in games that are monetized differently. Also, one could argue that there could be other relevant factors that could have been beneficial for further understanding of the associations seen. One such factor could have been socio-economy that might influence a preference for a method of payment but also the risk for problematic gaming.

In the gaming world, some games are ‘viral’ and extremely popular for a short period of time (a recent example is Hogwarts Legacy). Given the everchanging nature of the field of video games and its variability over time, drawing definitive conclusions and comparing studies from different periods can be problematic. Another related issue is that some gaming communities could have overlap with other communities, in which one of the games could have a higher addiction potential and a GASA-score that ‘spills over’ into the other game.

## Conclusion

The categorizing of games into genres is increasingly complex and the result of this study suggests that the financial model could be a valid alternative explanatory model of GD symptoms. Perhaps, we thereby could avoid measuring multiple and divergent structural characteristics with an alternative, relevant and straightforward understanding of the pathological potential of different games. Also, future research would benefit from exploring not only *how* the participants play the games in addition to the titles and categories played but also what aspect or mechanism that holds the most addictive potential. Possibly, there are areas that could or *should* be regulated to reduce the negative consequences of gaming.

## Data Availability

The data that support the findings of this study are available from the corresponding author upon reasonable request.
